# Reciprocal Symbiont Sharing in the Lodging Mutualism between Walking Corals and Sipunculans

**DOI:** 10.1371/journal.pone.0169825

**Published:** 2017-01-10

**Authors:** Momoko Igawa, Hiroki Hata, Makoto Kato

**Affiliations:** 1 Graduate School of Human and Environmental Studies, Kyoto University, Sakyo, Kyoto, Japan; 2 Graduate School of Science and Engineering, Ehime University, Matsuyama, Ehime, Japan; UPMC, FRANCE

## Abstract

Solitary scleractinian corals of the genera *Heterocyathus* and *Heteropsammia* inhabit soft marine bottoms without attaching to hard substrata. The corallums of these genera contain a coiled cavity inhabited by a sipunculan that roams the seafloor, carrying the host coral with it. The coral serves as a sturdy shelter that protects the sipunculan from possible predators. At the same time, the sipunculan maintains the coral in an upright position on the soft bottom. This coral–sipunculan association is unique because two phylogenetically distant coral genera have developed convergent associations with sipunculans. We investigate the process of convergent evolution of two coral species, *Hc*. *aequicostatus* and *Hp*. *cochlea*, in Okinawa, Japan, with their symbiotic sipunculans, using phylogenetic and morphological analyses. Phylogenetic analyses clarified that the symbiotic sipunculans comprise two distinct clades, surprisingly both of which are associated with both coral species. The bodily habitus of the sipunculan differed between coral species and fit the morphologies of the coiled cavities of their respective host corals. Our results suggest that the two coral species share two sipunculan clades and that sipunculan morphology is plastic and determined by the internal structure of their host corals.

## Introduction

The scleractinian genera *Heterocyathus* and *Heteropsammia* are solitary corals that live on soft marine bottoms without attaching to hard substrata. They have received special attention because their corallums contain a coiled cavity inhabited by a sipunculid worm [1–5]. Observations on internal corallum structure suggest that a larval coral initially settles on a small gastropod shell that has already been colonized by a sipunculan, and that the coral grows over and ultimately beyond the shell, providing a coiled cavity for the equally growing worm partner. The coral provides a sturdy shelter, protecting the worm against possible predators. Furthermore, the worm can roams the seafloor, dragging its host coral along [6–9]. If the coral is overturned by water currents or buried by sedimentation, the worm contributes to the coral’s recovery to an upright position. Thus, the association between coral and sipunculan has been hypothesized to be mutualistic [10–11].

Irrespective of their similarities in morphology and ecology, the two coral genera are classified into separate families and suborders [12]: *Heterocyathus* in the family Caryophylliidae (suborder Caryophylliina) and *Heteropsammia* in the family Dendrophylliidae (suborder Dendrophylliina). Thus, the coral—sipunculan association is an example of convergent coevolution.

However, it has not been ascertained whether the sipunculans inhabiting different coral genera belong to a single species. Although Bouvier [2] argued that the sipunculans associated with the two coral genera are different species, others have suggested that they are the same species [8, 13], *Aspidosiphon muelleri* Diesing, 1851 [14]. *A*. *muelleri* is a wide-spread, eurytopic, and polymorphic species that has a long list of junior synonyms [15–16], and is known to inhabit empty gastropod shells (occasionally scaphopod shells or polychaete tubes). Improved understanding of host specificity of sipunculans symbiotic with these two coral genera could shed light on how the convergence of coral-sipunculan symbiosis evolved in history.

The neritic sandy bottom in Kin Bay, Okinawa, Japan is inhabited by two walking coral species, *Hc*. *aequicostatus* and *Hp*. *cochlea*. To elucidate the process of convergent evolution for these two coral genera, we first examined the corals’ internal morphology and the bodily habitus of the symbiotic sipunculans, and then analysed the sipunculans’ species identity using phylogenetic analyses.

## Materials and Methods

### Coral sampling

We sampled walking corals in Kin Bay, Okinawa, Japan on February 28, 2014. Permits for the research and collection of solitary corals were obtained from Kin-Nakagusuku Port and Okinawa prefecture, respectively. We dredged shallow soft bottoms (26°23.32'N, 127°52.52'E, 10–15 m deep) using a small dredge (RIGO, Tokyo, Japan; aperture rim size = 40 × 15 cm, mesh size = 2 mm). The dredged sediment contained 40 *Hc*. *aequicostatus* and 304 *Hp*. *cochlea* corallums, all of which were photographed. Of the corallums, 30 corallums for each species were fixed in 99% ethanol and brought back to our laboratory for analyses of DNA and internal corallum structure. At the dredging site, we also observed the bottom environment along with living corals directly via scuba diving, collecting several additional corals.

Furthermore, we collected additional corals by dredging soft bottoms of Oshima Strait (28°12.44'N, 129°14.23'E, 60–80 m deep), Amami Oshima Island, Kagoshima prefecture and Tosa Bay (33°16.46'N, 133°36.19'E, about 130 m deep), Kochi Prefecture, Japan. In these locations, no specific permissions were required and all corals of the genera *Heterocyathus* and *Heteropsammia* are not endangered or protected species. We collected one specimen of *Hc*. *alternatus* and one specimen of *Hp*. *cochlea* in Oshima strait and one specimen of *Hc*. sp. in Tosa Bay. The collected specimens were fixed in 99% ethanol and deposited in our laboratory.

### Observation and measurement of coral morphology

We measured the corallum size (length, greater diameter of corallum; width, lesser diameter of corallum; and height) of the collected corals. We used Pearson’s product-moment correlation coefficient to detect any correlations between coral length and width/height.

We observed the morphology of the coiled cavities of several corals by taking soft X-ray photographs using a C-60 X-ray inspection device (SOFTEX, Kanagawa, Japan; the applied voltage and exposure time were 50 kV and 2–4 min, respectively).

### Observation of the morphology of symbiotic sipunculans

To determine the presence/absence of sipunculans inhabiting corallum cavities, we cracked the ethanol-fixed corals open using a vise and collected any sipunculans without damaging them. Before vising a coral, we wrapped it in cling film to prevent pieces from scattering. Then, the corallum was pressed laterally in the vise until it cracked. If a sipunculan was found in the coral, we removed it with tweezers.

We took photographs of individual sipunculans and measured their body lengths and widths using the ImageJ analysis software (US National Institutes of Health, Bethesda, MA, USA). Because sipunculans have curved bodies, we measured the axis of the trunk to determine body length. We used Pearson’s product-moment correlation coefficient to detect any correlations between corallum size and sipunculan size.

### Phylogenetic analyses of sipunculans

We barcoded five gene regions of 16 sipunculans living in the two coral genera, *Heterocyathus* and *Heteropsammia*. DNA was extracted from a piece of tissue from the body wall or retractor muscle, following a standard CTAB protocol. We sequenced the fragments of two nuclear ribosomal genes (18S rRNA and 28S rRNA); one nuclear protein-coding gene (histone H3); one mitochondrial ribosomal gene (16S rRNA); and one mitochondrial protein-coding gene (cytochrome c oxidase subunit I, COI). Polymerase chain reactions (PCRs) were performed in 25 μl mixtures consisting of 0.4 μM of forward and reverse primers (for primer sequences, see S1 Appendix), 2.0 μl of dNTP, 2.5 μl of ExTaq buffer, 0.125 μl of ExTaq polymerase (TaKaRa, Otsu, Japan), 20.0 μl of distilled water, and 1 μl of template. Thermal cycling was performed with an initial denaturation for 5 min at 94°C, followed by 30 cycles of 30 s at 94°C, 30 s at a gene-specific annealing temperature (S1 Appendix), and 1 min at 72°C, with a final 7 min extension at 72°C. Sequence reactions were performed in 5 μl volumes, using 0.5 μl of BigDye^®^ Terminator (ver. 3.1; Applied Biosystems, Foster City, CA, USA), 0.16 μM of PCR primer, 1.5 μl of sequencing buffer, and 2.5 μl of template. They were electrophoresed on an ABI 3130 sequencer (Applied Biosystems).

The obtained sequences were aligned using ClustalW as implemented in the software MEGA 5.2 [17] under default settings. The resulting dataset contained 470, 309, 288, 516 and 454 base pairs of 18S rRNA, 28S rRNA, H3, 16S rRNA and COI, respectively. For phylogenetic analyses, we also included sequence data for other *Aspidosiphon* and Sipuncula taxa that were obtained from Kawauchi *et al*. [18].

We constructed phylogenetic trees by maximum-likelihood (ML) and Bayesian methods, using the programs Treefinder [19] and MrBayes (v. 3.2.5; [20]), respectively. Model selection of each analysis was conducted using Kakusan 4 [21]. The criteria used for model selection were AICc4 for ML analysis and BIC4 for Bayesian analyses. For ML analysis, K80+G, TN93+G, and GTR+G models were selected as the best-fit models for the 18S, 28S, and 16S genes, respectively. JC69 model was best fit for the first, second, and third codon positions of H3. We selected K80+G, F81+G, and TN93+G models for the first, second and third codon positions of COI, respectively. For Bayesian analyses, K80+G, TN93+G, and J1+G models were selected as the best-fit models for the 18S, 28S, and 16S genes, respectively. JC69+G, JC69, and J2+G models were best-fit for the first, second, and third codon positions of H3, respectively. We selected J2ef+G, F81+G, and TN93+G models for the first, second and third codon positions of COI, respectively. For both ML and Bayesian analyses, a gene proportional and codon proportional mixed model was selected. The robustness of the ML tree was validated by bootstrap analysis with 1000 replications. The Bayesian analyses consisted of running two simultaneous chains for 1,000,000 generations, sampling trees every 100 generations, for a total of 10,001 trees. Convergence diagnostics were assessed using TRACER v1.6 [22]. The initial 1,001 generations were discarded as burn-in. The analyses reached stability well before the burn-in period.

## Results and Discussion

**Fig 1:**
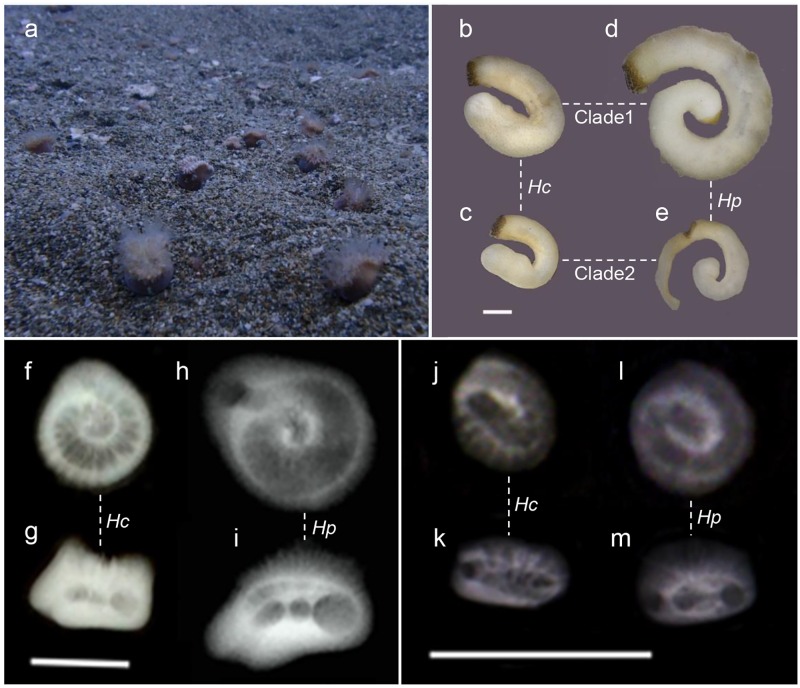
The habitat and specimens of studied walking corals and their symbiotic sipunulans. a, A dense population of walking corals on the sandy bottom of Kin Bay; b–e, Photographs of ethanol-fixed sipunculans from clade 1 (b, d) and clade 2 (c, e) (scale bar = 1 mm), symbiotic with *Heterocyathus aequicostatus* (b–c) and *Heteropsammia cochlea* (d–e); f–m, Soft X-ray images of *Heterocyathus aequicostatus* (f–g, j–k) and *Heteropsammia cochlea* (h–i, l–m) of Kin Bay (scale bars = 1 cm), horizontal (f, h, j, l) and lateral (g, i, k, m) views of mature (f–i) and immature (j–m) individuals.

We found that two species of walking corals, *Hc*. *aequicostatus* and *Hp*. *cochlea*, co-occurred densely on the sandy bottom of Kin Bay at depths of 10−15 m (Fig 1a). Most corals were found with their lower portions embedded in the sediment. By dredging the bottom of Kin Bay (about 115 m^2)^, we found 40 specimens of *Hc*. *aequicostatus* and 304 specimens of *Hp*. *cochlea* (see S2 Appendix). Additionally, we collected one specimen of *Hc*. *alternatus* and one specimen of *Hp*. *cochlea* in Oshima strait and one specimen of *Hc*. sp. in Tosa Bay.

There were positive correlations between the length and the width of the corallum in both coral species (*Hc*. *aequicostatus*, N = 43, r = 0.968, p < 0.001; *Hp*. *cochlea*, N = 304, r = 0.969, p < 0.001). Similar correlations were also detected between the length and the height of the corallum (*Hc*. *aequicostatus*, N = 43, r = 0.967, p < 0.001; *Hp*. *cochlea*, N = 304, r = 0.951, p < 0.001; see S3 Appendix).

Soft X-ray observation (Fig 1f–1m) suggested that the corallum cavity coils horizontally in young individuals and then descends steeply downward as the coral matures in both coral species. However, the coil trajectory of the corallum cavity differed among the coral species. In addition, the coiled cavities of *Hc*. *aequicostatus* were thicker than those of *Hp*. *cochlea*, especially among smaller corals.

**Fig 2:**
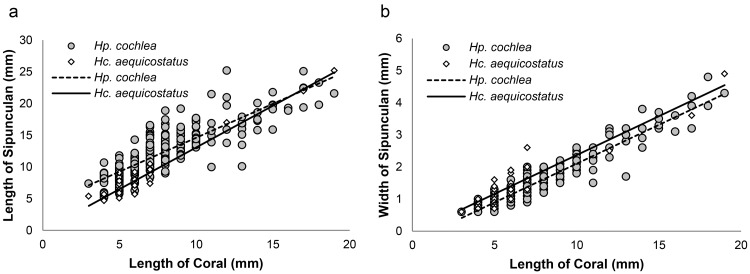
Relationships between corallum size and sipunculan size. Relationships between corallum length of the host corals and length (a) and width (b) of their symbiotic sipunculans in *Heterocyathus aequicostatus* and *Heteropsammia cochlea*.

We cracked a part of 40 and 304 corallum specimens of *Hc*. *aequicostatus* and *Hp*. *cochlea*, respectively, and found that the association between the corals and sipunculans was obligate, as almost all cracked corals (95% of *Hc*. *aequicostatus* and 100% of *Hp*. *cochlea*) were inhabited by sipunculans. These sipunculans filled the entire width of the corallum cavity that they inhabited, coiling their bodies in the same manner as their host’s cavity. *Hc*. *aequicostatus* without sipunculans also had coiled cavities in their corallums, so it is likely that their symbiotic sipunculans had existed at first, but died or left later. We found positive correlations between the corallum length and the sipunculan’s length in both coral species (*Hc*. *aequicostatus*, N = 38, r = 0.965, p < 0.001; *Hp*. *cochlea*, N = 287, r = 0.848, p < 0.001; Fig 2a). Multiple linear regression analyses showed that the slopes of the two regression lines were significantly different (F_3, 321_ = 368.4, t = –2.284, p < 0.05), and that the intercepts of these lines were also significantly different (t = 5.159, p < 0.001). Positive correlations were also detected between the corallum length and the width of the resident sipunculan (*Hc*. *aequicostatus*, N = 38, r = 0.925, p < 0.001; *Hp*. *cochlea*, N = 287, r = 0.952, p < 0.001; Fig 2b), and the intercepts of the two regression lines were again significantly different (F_3, 321_ = 958.3, t = –2.684, p < 0.01). These data suggest that the corals and the sipunculans grow at similar rates, and that sipunculans living in *Hc*. *aequicostatus* are relatively thicker, shorter, and less coiled than those living in *Hp*. *cochlea*, especially while they are immature. In the case of the sipunculan *Aspidosiphon muelleri*, which inhabits polychaete tubes, the sipunculan grows until it completely fills the tube, and then moves to a larger tube [23]. The sipunculans symbiotic with walking corals live in coiled coral cavities that expand simultaneously with the sipunculans’ growth, so they need not move to larger lodgings. This coral—sipinculan association can be viewed as lodging mutualism.

**Fig 3:**
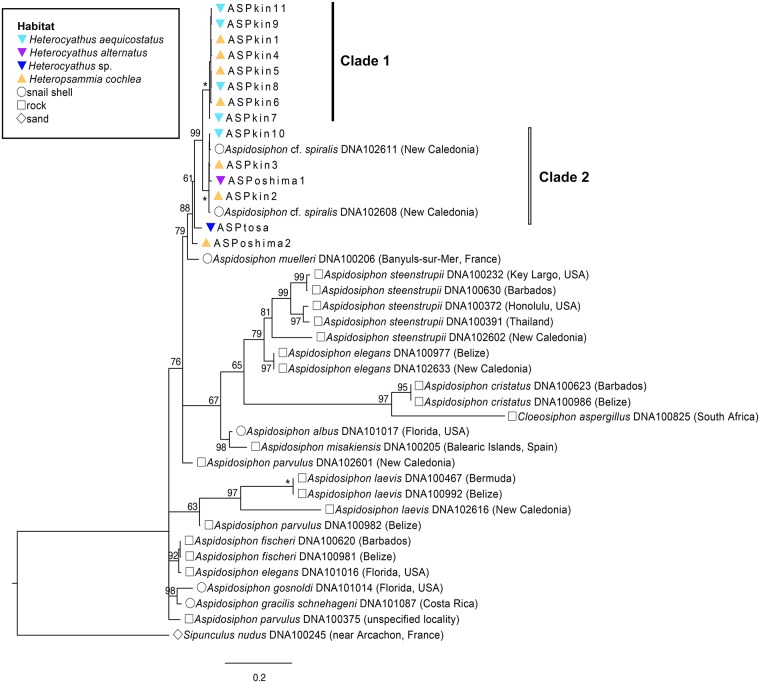
Maximum-likelihood tree of *Aspidosiphon* sipunculans based on the combined data set of five gene regions. Numbers on nodes indicate maximum-likelihood bootstrap support values. Asterisks correspond to BS = 100%. ASPkin1–11, ASPoshima1–2, and ASPtosa are sample numbers given to the specimens collected from Kin Bay, Oshima Strait, and Tosa Bay, respectively. Each symbol to the left of species names indicates sipunculan domicile. The accession number and the provenance of each specimen are also shown.

**Fig 4:**
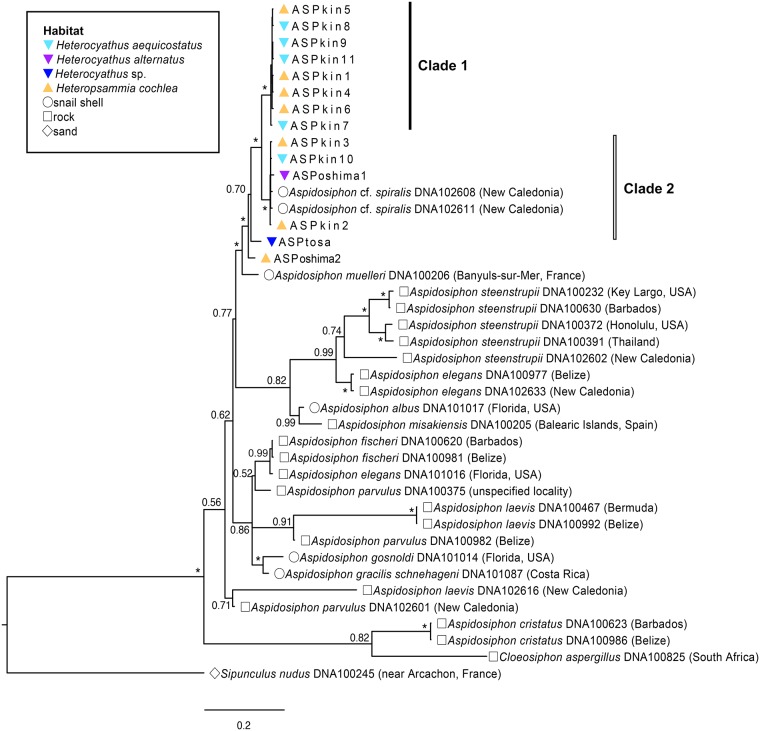
Bayesian tree of *Aspidosiphon* sipunculans based on the combined data set of five gene regions. Numbers on nodes indicate Bayesian posterior probabilities. Asterisks correspond to PP = 1.00. ASPkin1–11, ASPoshima1–2, and ASPtosa are sample numbers given to the specimens collected from Kin Bay, Oshima Strait, and Tosa Bay, respectively. Each symbol to the left of species names indicates sipunculan domicile. The accession number and the provenance of each specimen are also shown.

The sipunculans living in walking corals clearly belonged to the genus *Aspidosiphon* because they had chitinous shield-like structures at the anterior and posterior extremities of their trunks, a key feature characterizing this genus. Phylogenetic trees (Figs 3 and 4) surprisingly revealed that the symbiotic sipunculans in Kin Bay comprise two distinct clades (clade 1 and clade 2), both of which are associated with two coral species. At this point, it is difficult to conclude that the two sipunculan clades represent different species because genetic distance between these two clades was less than intraspecific divergence of known *Aspidosiphon* species (e.g., *A*.*steenstrupii*). These results suggest that the association of sipunculan with host coral species is not species-specific, and that a single sipunculan species that involves two clades or two distinct sipunculan species are shared by the two host coral species. In Oshima Strait and Tosa Bay, two rare *Heterochyathus* species, *Hc*. *alternatus* and *Hc*. sp., were collected respectively. The sipunculan associated with the former coral species belonged to the clade 2, and the sipunculan associated with the latter was in another clade (Figs 3 and 4). Our results suggest that sipunculans symbiotic with walking corals are more diverse than expected, and that the association between the corals and the sipunculans might have experienced multiple host-switching as well.

Though we found no intrinsic morphological differences between the two sipunculan clades, the morphology of the sipunculans differed distinctly between coral species (Fig 1b–1e). As a result, the structure of the coiled cavities constrains the morphology of the inhabitant sipunculans, suggesting that sipunculan morphology is plastic and determined by the internal structure of their host corals.

## Supporting Information

S1 Appendix Information on primer sequences and PCR conditions. (PDF)

S2 Appendix Table of specimens found by dredging the bottom of Kin Bay. (XLSX)

S3 Appendix Relative growth of corallum. Relative growth of corallum in *Heterocyathus aequicostatus* and *Heteropsammia cochlea*. (a) Relationships between length and width of corallum. (b) Relationships between length and height of corallum.
(PDF)
