# Supplementary material for: Reciprocal Symbiont Sharing in the Lodging Mutualism between Walking Corals and Sipunculans
Source: PLoS One. 2017 Jan 10;12(1):e0169825. doi: 10.1371/journal.pone.0169825 (PMC5224867; doi:10.1371/journal.pone.0169825)
Supplement: S1 Appendix — (PDF) [file pone.0169825.s001.pdf]

S1 Appendix: Information on primer sequences and PCR conditions.

Primer	Sequence5'– 3'	PCR condition
18S rRNA		
18S-H17F [1]	AAA TTA CCC ACT CCC GGC A	94°C 5 min, (94°C 30 s, 50°C 30 s,
18S-H35R [1]	TGG TGA GGT TTC CCG TGT T	72°C 1 min) × 30, 72°C 7 min
28S rRNA		
28Sa [2]	GAC CCG TCT TGA AAC ACG GA	94°C 5 min, (94°C 30 s, 48°C 30 s,
28Sb [2]	TCG GAA GGA ACC AGC TAC TA	72°C 1 min) × 30, 72°C 7 min
H3		
H3aF [3]	ATG GCT CGT ACC AAG CAG ACV GC	94°C 5 min, (94°C 30 s, 52°C 30 s,
H3aR [3]	ATA TCC TTR GGC ATR ATR GTG AC	72°C 1 min) × 30, 72°C 7 min
16S rRNA		
16Sa [4]	CGC CTG TTT ATC AAA AAC AT	94°C 5 min, (94°C 30 s, 45°C 30 s,
16Sbr [5]	CCG GTT TGA ACT CAG ATC ATG	72°C 1 min) × 30, 72°C 7 min
COI		
LCO1490 [6]	GGT CAA CAA ATC ATA AAG ATA TTG G	94°C 5 min, (94°C 30 s, 45°C 30 s,
HCO2198 [6]	TAA ACT TCA GGG TGA CCA AAA AAT CA	72°C 1 min) × 30, 72°C 7 min

Additional references

1. Heraty J, Hawks D, Kostecki JS, Carmichael A. Phylogeny and behaviour of the Gollumiellinae, a new subfamily of the ant-parasitic Eucharitidae (Hymenoptera: Chalcidoidea). Syst Entomol. 2004;29: 544–559. doi: 10.1111/j.0307-6970.2004.00267.x
2. Whiting MF, Carpenter JM, Wheeler QD. The Strepsiptera problem: phylogeny of the holometabolous insect orders inferred from 18S and 28S ribosomal DNA sequences and morphology. Syst Biol. 1997;46: 1–68. doi: 10.1093/sysbio/46.1.1
3. Colgan DJ, McLauchlan A, Wilson GDF, Livingston SP, Edgecombe GD, Macaranas J, et al. Histone H3 and U2 snRNA DNA sequences and arthropod molecular evolution. Aust J Zool. 1998;46: 419–437. doi: 10.1071/ZO98048
4. Xiong B, Kocher TD. Comparison of mitochondrial-DNA sequences of 7 morphospecies of black flies (Diptera, Simuliidae). Genome. 1991;34: 306–311.
5. Xiandong D, Chen Z, Deng YW, Wang GH, Huang RL. Genetic diversity and population structure of the peanut worm (*Sipunculus nudus*) in southern China as inferred from mitochondrial 16S rRNA sequences. Isr J Aquacult-Bamid. 2008;60: 237–242. doi: 10.1139/g91-050

6. Folmer O, Black M, Hoeh W, Lutz R, Vrijenhoek R. DNA primers for amplification of mitochondrial cytochrome c oxidase subunit I from diverse metazoan invertebrates. Mol Mar Biol Biotechnol. 1994;3: 294–295.
