# Supplementary material for: Reciprocal Symbiont Sharing in the Lodging Mutualism between Walking Corals and Sipunculans
Source: PLoS One. 2017 Jan 10;12(1):e0169825. doi: 10.1371/journal.pone.0169825 (PMC5224867; doi:10.1371/journal.pone.0169825)
Supplement: S3 Appendix — Relative growth of corallum in Heterocyathus aequicostatus and Heteropsammia cochlea. (a) Relationships between length and width of corallum. (b) Relationships between length and height of corallum. (PDF) [file pone.0169825.s003.pdf]

S3 Appendix: Relative growth of corallum.

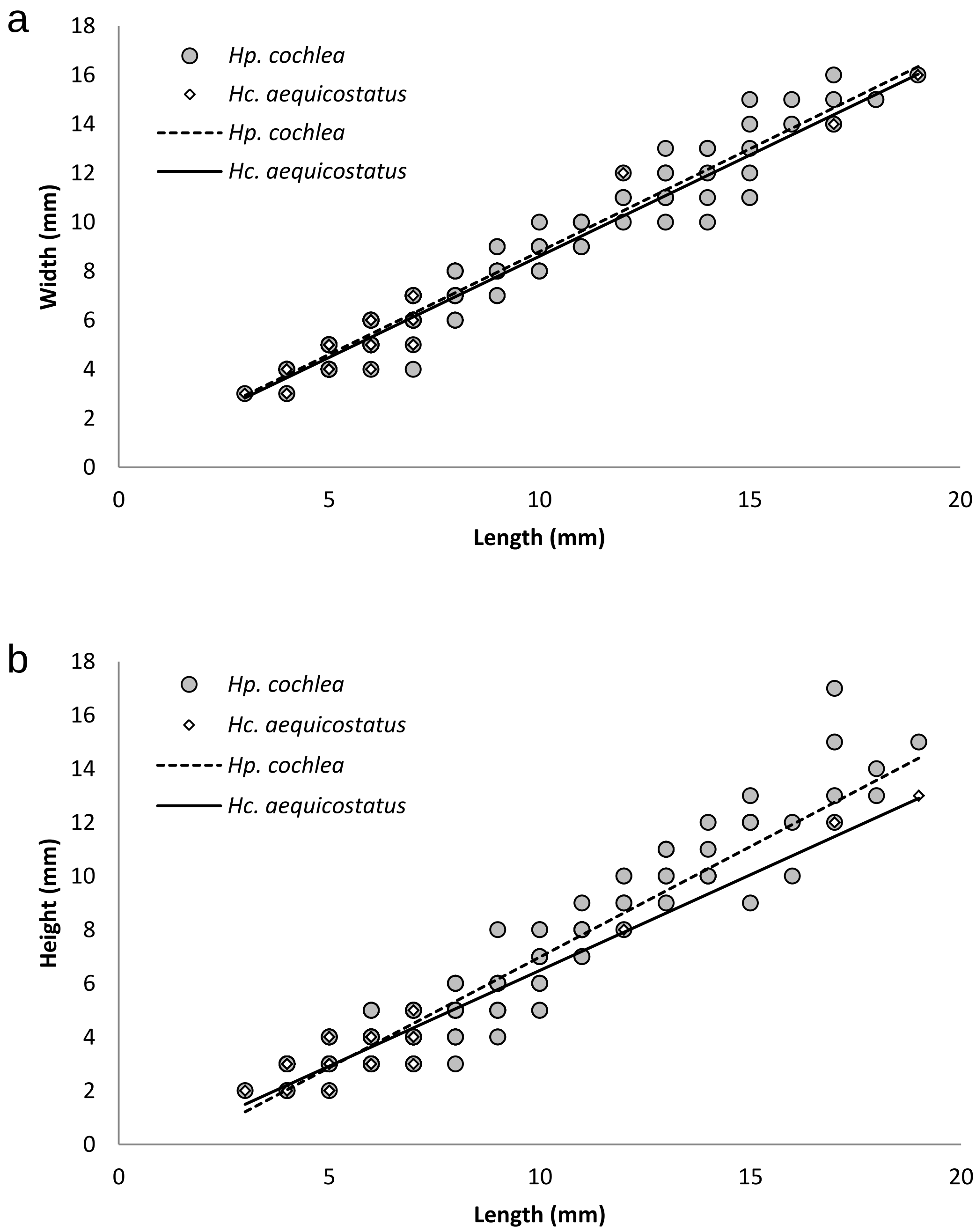

S3 Appendix. Relative growth of corallum in *Heterocyathus aequicostatus* and *Heteropsammia cochlea*. (a) Relationships between length and width of corallum. (b) Relationships between length and height of corallum.
